# Personalized Type 2 Diabetes Management Using a Mobile Application Integrated with Electronic Medical Records: An Ongoing Randomized Controlled Trial

**DOI:** 10.3390/ijerph18105300

**Published:** 2021-05-16

**Authors:** Eun-Young Lee, Jae-Seung Yun, Seon-Ah Cha, Sun-Young Lim, Jin-Hee Lee, Yu-Bae Ahn, Kun-Ho Yoon, Seung-Hyun Ko

**Affiliations:** 1 Division of Endocrinology and Metabolism, Department of Internal Medicine, Seoul St. Mary’s Hospital, College of Medicine, The Catholic University of Korea, Seoul 06591, Korea; leyme@catholic.ac.kr (E.-Y.L.); yoonk@catholic.ac.kr (K.-H.Y.); 2 Division of Endocrinology and Metabolism, Department of Internal Medicine, St. Vincent’s Hospital, College of Medicine, The Catholic University of Korea, Seoul 16247, Korea; dryun@catholic.ac.kr (J.-S.Y.); kakki@catholic.ac.kr (S.-A.C.); ybahn@catholic.ac.kr (Y.-B.A.); 3 Institute of Catholic Ubiquitous Health Care, The Catholic University of Korea, Seoul 06591, Korea; sun6309@catholic.ac.kr (S.-Y.L.); jheelee@catholic.ac.kr (J.-H.L.)

**Keywords:** type 2 diabetes mellitus, digital health, mHealth, self-monitoring

## Abstract

Controlling type 2 diabetes (T2DM) requires a comprehensive approach including patient education, self-monitoring of blood glucose, individualized behavioral strategies, and frequent contact with healthcare professionals (HCPs). We aimed to compare the efficacy of a personalized lifestyle intervention based on a mobile phone application with regular care in participants with T2DM. This is an ongoing randomized controlled open-label parallel-group trial with a target accrual of 282 participants, of which 181 have been enrolled to date. Participants are randomly assigned to one of three groups: (1) regular care; (2) mobile diabetes management; or (3) mobile diabetes management with HCP feedback. The mobile application is enabled to integrate with both electronic medical records (EMR) and a web-based diabetes management system for HCPs. It can send customized messages based on participants’ responses to lifestyle questionnaires administered at the baseline. The intervention period is 26 weeks followed by observation for 26 weeks. We evaluate the intervention’s features in order to assess its clinical utility and efficacy and compare outcomes with regular care considering relevant clinical factors, such as age, baseline HbA1c, etc. We expect our study to provide new evidence in support of customized mobile application tools for the management of T2DM.

## 1. Introduction

Diabetes mellitus is a major health concern affecting 463 million people worldwide [1]. Due to related complications and comorbidities, an estimated 4.2 million deaths were attributed to diabetes in 2019 [1,2]. Intensive glycemic control can reduce morbidity and mortality by decreasing micro- and macrovascular complications [3]. However, to achieve glycemic goals, in addition to medical treatment, self-management strategies, including diet, exercise, and self-monitoring of blood glucose (SMBG), are essential. Its complex nature challenges patients and providers to properly manage diabetes, with only 25.1% of patients meeting the glycated hemoglobin (HbA1c) target of <6.5% and 52.6% meeting the HbA1c target of <7.0% in Korea [4]. Furthermore, only 8.4% of patients with diabetes met combined glycemic, lipid, and blood pressure goals [4]. In the United States, 37.0% and 7.3% of patients, respectively, met the HbA1c target of <7.0% and combined glycemic, lipid, and blood pressure goals [5]. Taken together, these data indicate how difficult it is to achieve target glycemic goals despite remarkable advances in anti-diabetes medications.

In response to this concern, mobile applications have recently emerged as a promising technology to enable effective self-management of diabetes. These tools can aid patients with diabetes by increasing awareness of and modifying behavior through reminder and/or feedback [6]. Such applications allow remote assessment and prompt intervention by healthcare professionals (HCPs) between visits, especially in urgent situations, such as hypoglycemia or severe hyperglycemia [7]. Mobile applications have been reported as superior to interventions such as text messaging, mobile device use, or conventional self-management. Several meta-analyses have shown that use of mobile applications can reduce HbA1c levels by 0.5%, with an even greater reduction when combined with HCP feedback [7,8,9].

Notwithstanding, several limitations have been observed in previous studies of mobile applications. Interventions were heterogeneous, not individualized, and not compared with simple interventions such as regular calls or automated alert messages [10,11]. Clinical effects varied among interventions and were generally modest [10]. Although the important role of HCPs is well understood, it has not been evaluated simultaneously with mobile applications and, while several studies evaluated cost effectiveness, the results were inconclusive [12,13].

**Figure 1:**
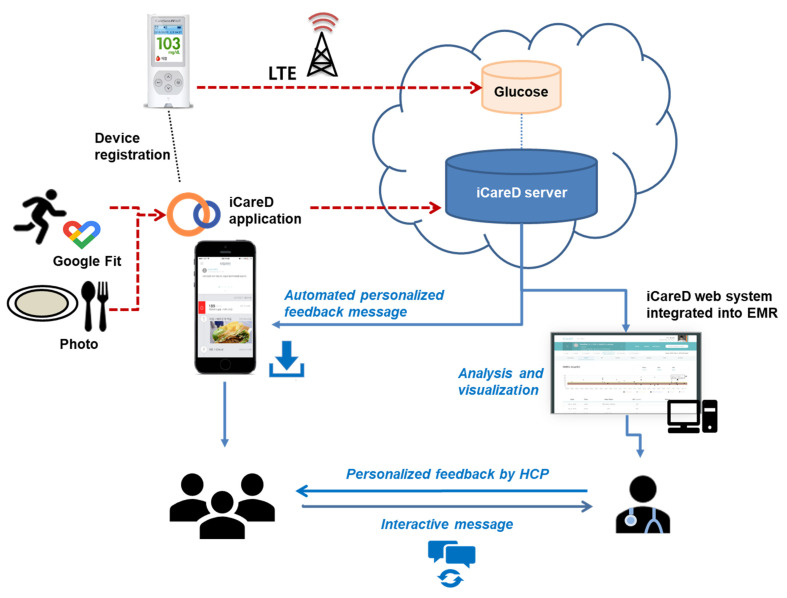
Technical architecture of the health coaching system supported by remote patient monitoring. EMR: electronic medical record; HCP: healthcare professional; LTE: long-term evolution. Broken bars represent raw data. Red and blue mean input and output, respectively.

To address these weaknesses, we designed a diabetes management system using an electronic medical record (EMR)-integrated mobile application to support type 2 diabetes (T2DM) management (Figure 1). The purpose of this study is to evaluate the utility, clinical efficacy, and cost effectiveness of a personalized diabetes management system (with and without HCP feedback) using an EMR-integrated mobile application in participants with T2DM compared with regular care. 

## 2. Materials and Methods

### 2.1. Study Design

**Figure 2:**
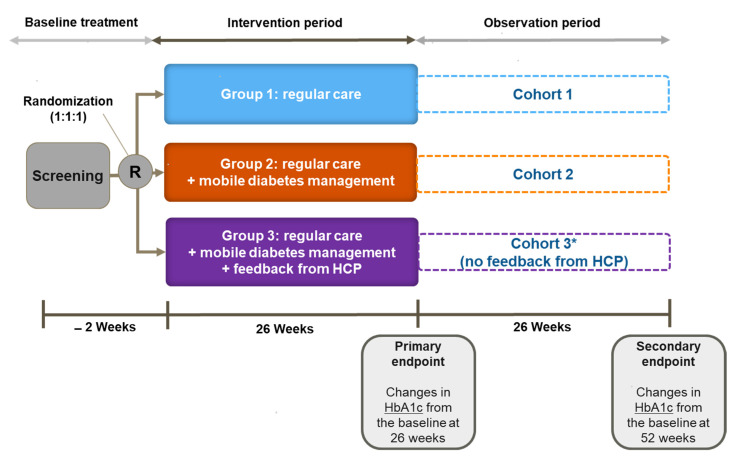
Schematic diagram of the study protocol. HCP: healthcare professional; R: randomization. * No feedback from HCP in Group 3 during the observation period.

This study is an open-label parallel-group three-arm randomized controlled trial conducted at two clinical study sites in Seoul, Korea: St. Vincent’s Hospital and St. Mary’s Hospital. The trial was registered at https://cris.nih.go.kr/cris/index.jsp (accessed on 1 January 2020), Clinical Research Information Service (CRIS), Republic of Korea (No. KCT0004128). Eligible participants are randomly assigned to one of the following three groups: (1) regular care; (2) mobile diabetes management; or (3) mobile diabetes management with personalized HCP feedback (Figure 1 and Figure 2). The participants are seen every 12 weeks ± 2 weeks. This trial is composed of two phases: a 26-week intervention period and a subsequent 26-week observation period. During the intervention period, the participants are managed according to the relevant protocols. In the subsequent observation period, the participants are managed the same as during intervention but without HCP feedback in Group 3 (Figure 2). This trial is ongoing; the first participant was enrolled in August 2019, and the final participant will be observed through December 2021.

### 2.2. Intervention

Regardless of the assigned group, all the participants are provided a glucometer (CareSens^®^N IoT, i-SENS, Inc., Seoul, Korea) during the intervention period along with diabetes education including SMBG, diet, and exercise.

The participants in Group 1 (the control group) receive regular care according to the Korean Diabetes Association standards [14]. They are instructed to conduct SMBG four times/day (before the morning meal and 2 h after every meal) and record it. Providers monitor SMBG at every visit and provide regular care (Table 1).

**Figure 3:**
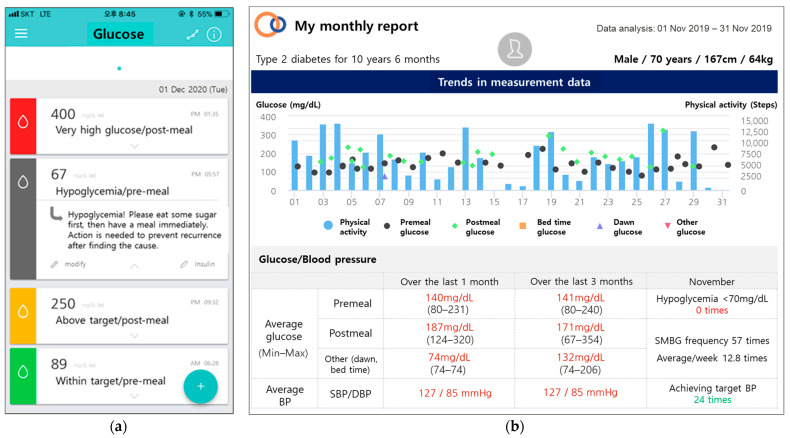
Examples of visual information of glycemic status (**a**) and a monthly report (**b**) on a web-based diabetes management system integrated with a mobile application.

In Groups 2 and 3 (intervention groups), the diabetes management system (iCareD application, Medical Excellence Inc., Seoul, Korea) is used in addition to regular care (Table 1). The instruction for SMBG is the same for all the groups; however, as glucometers transmit data wirelessly through mobile communication technology (long-term evolution), the participants in Groups 2 and 3 can track their SMBG data using the application without manual recording. Further, they can visually track the glycemic status as the glucose level is differentiated by color in the application (Table 2). For example, grey signifies potential hypoglycemia; green indicates a glycemic level within the target range; and red indicates a very high glucose level (Table 2, Figure 3A). The participants are also instructed to upload diet photos through the application. Physical activity is tracked using the Google Fit^®^ mobile application [15]. These data are uploaded to the web-based diabetes management system which is integrated to the EMR system for HCP use. At every visit, providers individualize the intervention based on the data. The participants in Groups 2 and 3 also receive messages about diabetes management through the application three times/week (two standardized messages for diabetes management and lifestyle modification and one customized message depending on the lifestyle questionnaire completed at the baseline). The messages consist of information on general diabetes management, diet, physical activity, and encouragement for self-care (Supplementary Table S1). The messages were developed based on the standard diabetes care and were verified by an independent endocrinologist, nurse, nutritionist, and exercise therapist. The participants in Groups 2 and 3 also receive a monthly report generated from their personal data (glucose, blood pressure, physical activity, and diet) (Figure 3B).

In Group 3, providers remotely monitor the participants’ data between visits and provide additional feedback every two weeks. The messages generally reflect three areas: glycemic status, diet, and physical activity. The participants in Group 3 may directly message providers and receive immediate feedback as needed. Individual feedback between visits will be provided only during the intervention period. There is no difference in intervention between Groups 2 and 3 during the observation period.

### 2.3. Eligibility

Eligible participants must meet all the inclusion criteria: (1) patients with T2DM who need SBMG and who underwent self-management training; (2) age of 19–74 years; (3) HbA1c ≥ 7.5%; (4) body mass index (BMI, calculated as kg/m^2^) ≥ 18.5 kg/m^2^; (5) ability to use a smartphone; and (6) written informed consent.

Exclusion criteria are: (1) insulin pump use; (2) estimated glomerular filtration rate < 30 mL/min/1.73 m^2^; (3) heart failure (New York Heart Association class III–IV); (4) diagnosis of cancer within five years; (5) recipient of an organ transplant or need in long-term immunosuppressant therapy; (6) difficulty in exercise and physical activity due to spinal disease (intervertebral disc prolapse, spinal stenosis, etc.), joint disease, or major surgery at the time of screening; (7) plan to receive surgery that could limit physical activity during the study period; (8) pregnant or breastfeeding; (9) plan to become pregnant or disagreement with using adequate contraception during the study period; (10) participation in another clinical trial (other than an observational one) within 90 days; and (11) inappropriate candidate as judged by an investigator.

### 2.4. Primary and Secondary Outcomes

The primary outcome is the change in HbA1c (%) from the baseline to week 26. The secondary outcome is the change in HbA1c (%) and fasting glucose (mg/dL) from week 26 to week 52. Other secondary outcomes include lifestyle changes based on physical activity and diet records; cardiometabolic risk factors (body weight, blood pressure, lipid profile); quality of life using Euro-QOL (EQ-5D-5L); participant satisfaction and adherence; number of unscheduled hospital visits due to hypoglycemia or hyperglycemia; number of hypoglycemia and hyperglycemia episodes; changes in the homeostasis model assessment for insulin resistance and β cell function (HOMA-IR and HOMA-β, respectively); and cost effectiveness.

Exploratory outcomes are the change in diabetes medication; frequency of SMBG; BMI; and albuminuria. Study results will be shared with participants and the larger medical research community through presentation at conferences and publication in peer-reviewed journals.

### 2.5. Measurements

Demographic, clinical, and lifestyle information collected at the baseline includes age; sex; duration of T2DM; alcohol consumption; smoking history; dietary habits; physical activity; other comorbidities; and medications, including anti-diabetes agents. Physical activities are assessed using the Korean version of the Global Physical Activity Questionnaire (K-GPAQ), a validated tool to assess the effectiveness of interventions promoting physical activity [16,17]. The lifestyle questionnaire administered at the baseline is repeated at week 26 and week 52. Anthropometrics such as height (m), body weight (kg), waist circumference (cm), and blood pressure (mmHg) are measured at every visit. Body composition data are obtained using a bioimpedance analyzer (InBody 720 and 970, InBody Co., Ltd. Seoul, Korea) at the baseline, week 26, and week 52. Laboratory parameters including fasting glucose, HbA1c, and lipid profile are collected at every visit. Serum fasting glucose is analyzed by hexokinase UV. Serum total cholesterol and triglycerides are measured by an enzymatic method, and high-density lipoprotein cholesterol is measured using a selective inhibition method. Low-density lipoprotein (LDL) cholesterol levels are estimated using the Friedewald equation: LDL cholesterol (mg/dL) = total cholesterol (mg/dL) − [triglycerides (mg/dL)/5] − high-density lipoprotein cholesterol (mg/dL) [18]. If the triglyceride level exceeds 400 mg/dL, the LDL cholesterol levels are directly measured using an enzymatic method. All the measurements are performed using a Hitachi 7600 autoanalyzer (Hitachi Instruments Service, Tokyo, Japan). EDTA plasma HbA1c is measured with high-performance liquid chromatography (Tosoh-G8, Tosoh, Tokyo, Japan). C-peptide and urinary albumin-to-creatinine is measured at the baseline and once every 26 weeks. Serum C-peptide is measured using an electrochemiluminescent immunoassay (E170, Roche Diagnostics, Indianapolis, USA). Spot urine albumin and creatinine are measured using a turbidimetric immunoassay and the Jaffe method, respectively. The updated homeostasis model assessment (HOMA2) calculator is used to evaluate HOMA-IR and HOMA-β [19,20]. The HOMA2 model has proven to be more predictive than the original HOMA1 model in Korean patients with T2DM [21].

Hypoglycemic events, including hospitalization or emergency room visits, are evaluated at every visit. Hypoglycemia is defined as blood glucose level < 70 mg/dL or when participants experience symptoms consistent with hypoglycemia, such as sweating, anxiety, or shakiness, and relief of those symptoms occurs following ingestion of glucose [22,23]. Diabetes management strategies, such as SMBG frequency, physical activity, and diet, are obtained at every visit. Quality of life is assessed using the Korean version of the Euro-QOL (EQ-5D-5L) instrument at the baseline and once every 26 weeks [24,25]. The EQ-5D-5L comprises five dimensions (mobility, self-care, usual activities, pain/discomfort, and anxiety/depression) rated on a scale of five (none, slight, moderate, severe, and extreme). User satisfaction with the mobile application will be surveyed in Groups 2 and 3 at the end of the study. Adherence will be assessed by evaluating the scheduled visits, SMBG frequency, maintenance of physical activity and diet record, and achievement of the HbA1c goal. Any adverse events occurring during the trial will be monitored until resolution and reported as required.

### 2.6. Sample Size Calculations

Based on the previous studies [7,8,26], we assumed a mean difference in the HbA1c level of at least 0.60 between the control and intervention groups and a standard deviation within a group of 0.75 after six months. In a single-factor analysis of variance (ANOVA), it was determined that a sample size of 219 (73 per group) would yield 90% power and a 0.05 significance level. We planned both an interim and a final analysis. Therefore, RB = (2, 0.05, 0.1) = 1.007 was applied as the O’Brien–Fleming test. Finally, a total of 282 subjects (94 per group) are required with a predictive dropout rate of up to 20% to achieve 73 subjects per group. This is an ongoing trial in which 181 participants have been enrolled to date.

### 2.7. Randomization

Randomization is performed to ensure scientific validity of the clinical trial by maximizing comparability between the groups and to prevent subjective involvement by investigators. An independent statistician having no contact with participants performs the randomization process. Eligible participants who provide written informed consent are randomly assigned to one of the three groups in a 1:1:1 ratio. Random numbers are generated using SAS version 9.3 (SAS Institute Inc., Cary, NC, USA) and sequentially numbered, opaque, sealed envelopes are used for allocation. Stratification is by study site and baseline HbA1c of 8.5% using the stratified permutated block randomization method. As this study is an open-label design, group assignment is known to the participants and HCPs after randomization.

### 2.8. Statistical Analysis

The data are collected using electronic case report forms and are password-protected. Only the principal investigator and authorized research personnel may access the data. Range checks on data values will be performed prior to analysis. Continuous variables will be presented as the means ± the standard deviation while categorical data will be presented as frequency and percentage. An analysis of covariance (ANCOVA) will be used to compare the mean HbA1c at week 26 between the three groups. The analysis will be conducted by adjusting for study site and baseline HbA1c of 8.5%. Post-hoc analysis will be performed with the Bonferroni method. The mixed-effect model repeated measures (MMRM) will be used to compare change in HbA1c from the baseline to week 26. For binary outcome variables, such as the rate of achieving the target HbA1c level, a generalized linear mixed model (logistic GLMM) will be used. The fixed effects in the MMRM and GLMM include treatment group indicators, time indicators, and interactions between the treatment group and time. The number of hypoglycemic events will be compared between the groups using the chi-squared or Fisher’s exact test.

Analysis will be performed as both per protocol and intention-to-treat. Unless otherwise specified, analyses will be reported based on the results of the intention-to-treat analysis. For interim analysis, adjustment for multiplicity will be applied. Based on the O’Brien–Fleming test, the significance level will be set at 0.005 and 0.0048 for interim and final analysis, respectively. Analyses will be performed using SAS version 9.3 (SAS Institute Inc., Cary, NC, USA).

### 2.9. Ethics

The study protocol was approved by the ethics committee of St. Vincent’s Hospital (IRB No. VC19EEDI0085) and St. Mary’s Hospital (IRB No. KC19EEDE0278) and is conducted in compliance with the Declaration of Helsinki. The providers introduce the trial to the patients and obtain informed consent. All the participants provide written informed consent prior to study enrollment/randomization and are informed that they may withdraw from the study at any time for any reason. All the study data are anonymized according to the Good Clinical Practice guidelines of the International Conference on Harmonization. The IRB will conduct internal audits upon completion of registration. Any modifications to the protocol that may affect study conduct will be reviewed and approved by the IRB prior to initiating any change.

## 3. Discussion

The importance of patient self-management of diabetes cannot be overstated [27,28]. According to the diabetes guidelines, all patients with diabetes should participate in self-management education and behavior modification such as nutritional therapy, physical activity, appropriate use of medication and insulin, prevention and treatment of hypoglycemia, and psychological wellness [29]. While continuous feedback and training to implement self-management are recommended, many providers have reported that sustained education and personalized lifestyle intervention are not carried out due to time constraints during visits and inability to monitor daily blood glucose, diet, and physical activity [30,31]. Coupled with the challenges posed to the providers, compliance of patients with self-management recommendations is often poor and represents another major barrier to diabetes management [32].

Some recent clinical trials have indicated that mobile applications can improve traditional diabetes management for patients with inadequate glycemic control [7,8,9]. Mobile applications facilitate collecting data on SMBG, physical activity (step counter), and diet habits (food picture) compared with conventional methods such as an SMBG note or 24-h dietary recall [8,33]. Notably, the data collection features of such applications enable HCPs to obtain reliable real-time information and provide tailored feedback. The applications also enable patients with diabetes to have interactive communication with HCPs without limitations due to time or location. Some applications also have the functionality to send automated messages to trigger behavioral changes or encourage self-management [7,34]. Moreover, the increasing use of smartphones (approximately 67% of the world population in 2019) lends itself to the development and implementation of mobile applications to manage chronic diseases [35].

Despite previous evidence suggesting the benefit of mobile applications to manage diabetes, concerns have been raised. The studies were heterogeneous and generally showed only moderate clinical efficacy [10]. Some studies reported enhanced effects with HCP feedback [7,8], while others found that bidirectional messages had a similar effect as unidirectional messages [36]. Most previous studies compared interventions with conventional treatment with only a few comparing them with other tools such as automated messages or pedometer use [10]. The previous mobile health studies reporting on cost effectiveness used interventions with text messaging, video or phone calls, or an online glucose record keeper rather than interactive communication or tailored HCP feedback [37].

Taking into account the growing need for effective diabetes management tools and limitations of previous studies, we designed a mobile application to support both patients and HCPs in this shared goal. The current randomized clinical trial compares regular care with the mobile application both with and without HCP feedback. The application is integrated with hospital EMRs and with a web-based diabetes management system for HCPs. One unique feature is its ability to send automated messages based on individual lifestyle questionnaires; the algorithms and messages were developed and validated by an independent endocrinologist, nurse, nutritionist, and exercise therapist.

This study has several limitations, chiefly, that it cannot be blinded as the interventions are obvious to participants. In addition, this trial includes only the participants with T2DM, which may limit generalizability to other types of diabetes. There could also be an age bias since older patients may be less likely to embrace mobile technology. 

## 4. Conclusions

Mobile-based intervention and interactive communication between patients and providers may improve diabetes outcomes by complementing conventional management strategies. We expect our study to provide more solid evidence of the utility, efficacy, and cost effectiveness of the mobile-based technology to manage chronic health conditions such as T2DM.

## Figures and Tables

**Table 1:** Comparison of intervention protocols for each group.

Components	Control	Intervention	
	Group 1	Group 2	Group 3
Education	Comprehensive management of diabetes, including self-care	Comprehensive management of diabetes, including self-care	Comprehensive management of diabetes, including self-care
Instruction	Conduct and record SMBG four times/day	Conduct and record SMBG four times/day, upload diet photos	Conduct and record SMBG four times/day, upload diet photos
Monitoring	SMBG and lifestyle * questionnaire, laboratory data	SMBG and lifestyle * log in the web-based system, individualized monthly reports about comprehensive management, laboratory data	SMBG and lifestyle * log in the web-based system, individualized monthly reports about comprehensive management, laboratory data
Intervention	Regular care only	Regular care with mobile diabetes management	Regular care with mobile diabetes management
Feedback from HCPs	During the visits (every 13 weeks)	During the visits (every 13 weeks)	During the visits (every 13 weeks) and between the visits (every two weeks) through the mobile application
Immediate intervention between visits	Not possible	Not possible	Possible
Interactive patient–physician communication between visits	No	No	Yes

* Diet and physical activities. HCP: healthcare professional; SMBG: self-monitoring blood glucose.

**Table 2:** Visual information of operational glycemic status on the mobile application in the intervention group.

Level	Assessment	Color Code	Glucose Level (mg/dL)				
			Preprandial	Postprandial	Bedtime	Dawn	Other
1	Severe hypoglycemia	Black ■	<50	<50	<50	<50	<50
2	Hypoglycemia	Dark grey ■	50–69	50–69	50–69	50–69	50–69
3	Potential hypoglycemia	Grey ■	70–79	70–89	70–89	70–79	70–79
4	Within the target	Green ■	80–130	90–180	90–139	80–130	80–130
5	Above the target	Yellow ■	131–179	181–249	140–249	131–179	131–179
6	High	Orange ■	180–249	250–349	250–349	180–249	180–249
7	Very high	Red ■	≥250	≥350	≥350	≥250	≥250

## Data Availability

The data are not available at this time as this is currently an ongoing trial.

## Supplementary Materials

The following are available online at https://www.mdpi.com/article/10.3390/ijerph18105300/s1, Table S1: Examples of automatic application messages for diabetes care.
