# Supplementary material for: Personalized Type 2 Diabetes Management Using a Mobile Application Integrated with Electronic Medical Records: An Ongoing Randomized Controlled Trial
Source: Int J Environ Res Public Health. 2021 May 16;18(10):5300. doi: 10.3390/ijerph18105300 (PMC8155840; doi:10.3390/ijerph18105300)
Supplement: Supplementary file 1 [file ijerph-18-05300-s001.zip › ijerph-1157825-supplementary.pdf]

International Journal of
Environmental Research and Public Health

MDPI

Table S1. Examples of automatic application messages for diabetes care.

Type	Examples
General diabetes management	Good morning. This is iCareD.<br>How are you dealing with stress?<br><br>Moderate stress can increase your ability to overcome challenges, but excessive stress can disrupt your mental and physical balance and cause a variety of health problems. Therefore, it is important to properly manage stress.<br><br>A stressful day! Forget confusing thoughts and focus on 3 tips!<br><br>❶ Meditate for 5 minutes<br>❷ Take a light walk<br>❸ Drinking teas such as chamomile and lavender are helpful for mental and physical stability
	Hello! This is iCareD.<br>Alcohol - Is it 'poison' to me?<br><br>▶ The correct answer is YES!!<br>Drinking alcohol increases caloric intake and weakens self-control. This leads to weight gain.<br><br>In addition, drinking alcohol after exercise or on an empty stomach may reduce liver function and increase the risk of hypoglycemia.<br><br>Why don't you try to stop drinking alcohol?<br><br>※ However, if you've already stopped drinking, keep going!
Diet	Hello, this is iCareD.<br>Are you doing well with a balanced diet?<br><br>▶ It is recommended to eat [rice + 1–2 plates of protein + at least 2 plates of vegetables] at each meal for even nutritional intake.<br>▶ For blood sugar management, be careful not to eat especially grain-oriented meals such as rice or noodles. When dining out, choose a balanced diet with vegetables rather than foods with high carbohydrate.<br>▶ Vegetables have a lot of fiber, so they make you feel full - and they are low in calories, which is helpful for weight control.
	Hello, this is iCareD.<br>Do you eat breakfast?

	▶ For stable blood sugar management, it is most important to eat 3 meals of a similar amount at approximately the same time each day. While you might be tempted to skip breakfast, it is an important meal because it replenishes nutrition expended during sleep.<br>▶ If you skip breakfast, you may crave snacks or foods high in calories or carbohydrates (sweets, bread, juice), which adversely affects blood sugar management and weight control.<br><br>Develop a habit of eating breakfast for your health.
Physical activity	Hello, this is iCareD.<br>Did you know that regular exercise helps improve immunity to disease?<br><br>Regular exercise stimulates the nervous system and activates the functioning of immune cells, helping to strengthen your ability to fight disease.<br><br>Aerobic exercise such as jogging, cycling, rope skipping, or swimming increases oxygen intake and benefits your cardiovascular system, which boost immunity!
	Hello! This is iCareD.<br>Does stretching help to control blood sugar?<br><br>Stretching cannot replace aerobic or strength exercise to control blood sugar levels - however -<br>If you sit for long periods of time, getting up, moving, and stretching every 30 minutes can help increase oxygen levels and maintain blood sugar.<br><br>Try light stretching every 30 minutes!
Encouragement for self-care	"Every man is the builder of a temple, called his body…."<br>- Henry David Thoreau -<br><br>What kind of temple do you want to build?<br><br>Do you want to play an active role in your health?<br>If so, continue practicing positivity and make good lifestyle choices.<br><br>iCareD will support you.
